# Metabolomic shifts in beef steers rotationally grazing toxic endophyte-infected tall fescue under fall conditions

**DOI:** 10.3389/fvets.2026.1785530

**Published:** 2026-05-15

**Authors:** Ignacio M. Llada, Jeferson M. Lourenco, Mikayla M. Dycus, Utsav Lamichhane, Matthew K. Ross, Garret Suen, Zachery R. Jarrell, Dean P. Jones, Nicholas S. Hill, Nikolay M. Filipov

**Affiliations:** 1 Interdisciplinary Toxicology Program, University of Georgia, Athens, GA, United States; 2 Department of Physiology and Pharmacology, University of Georgia, Athens, GA, United States; 3 Department of Animal and Dairy Science, University of Georgia, Athens, GA, United States; 4 Department of Comparative Biomedical Sciences, Center for Environmental Health Sciences, Mississippi State University, Mississippi State, MS, United States; 5 Department of Bacteriology, University of Wisconsin, Madison, WI, United States; 6 Department of Medicine, Division of Pulmonary, Allergy, Critical Care & Sleep Medicine, Emory University, Atlanta, GA, United States; 7 Department of Crop and Soil Science, University of Georgia, Athens, GA, United States

**Keywords:** *Epichloë coenophiala*, ergot alkaloids, metabolomics, toxic tall fescue, volatile fatty acid (VFA)

## Abstract

**Background:**

Fescue toxicosis (FT) results from ingestion of tall fescue infected with the ergot alkaloid (EA)-producing endophyte *Epichloë coenophiala*. While the vascular system is a major EA target, their biogenic amine-like properties can trigger wider physiological effects. This study used untargeted metabolomics and targeted volatile fatty acid (VFA) analysis to characterize EA-induced metabolic disruption in steers under rotational tall fescue grazing.

**Methods:**

18 steers grazed toxic (E+), novel (NE), or endophyte-free (E-) fescue pastures. After 14 days, groups switched diets (toxic to nontoxic and vice versa). Urine, saliva, plasma, rumen fluid (RF), and feces were collected. Untargeted high-resolution metabolomics (HRM) was performed on liquid matrices, and gas chromatography-mass spectrometry quantified VFAs in RF and feces.

**Results:**

Total and individual VFA increased in RF during E+ exposure and returned to baseline after removal. Discriminative analyses showed that E+ steers had a distinct metabolome, while previously exposed steers in period 2 resembled those never exposed. Pathway analysis revealed downward shifts in beta-oxidation, fatty acid and arachidonic acid metabolism in E+, whereas aromatic amino acids (e.g., tyrosine, tryptophan), branched-chain amino acids, vitamin B6, and carbohydrate (e.g., gluconeogenesis) pathways shifted upwards. Upward-shifted pathways were mainly amino acid-related (45%), and downward-shifted mostly lipid-related (60%). Several metabolites, including tyramine, methyltyramine, methoxytyramine, and dopamine, were discriminatory for E+. HRM detected clavine alkaloids, and lysergic acid derivatives in all matrices except plasma, rising and returning to baseline within 2 days of E+ exposure/removal.

**Conclusion:**

Grazing E+ disrupts metabolism in steers, shifting energy use from lipids toward amino acids and carbohydrates. The main detected EAs were clavine-type alkaloids and simple lysergic acid amides, not ergopeptines, suggesting extensive biotransformation. The EA dynamics and the similar metabolic profile of previously and never-exposed steers in period 2, indicate minimal or no lasting effects metabolomic after exposure ends. The upward shift in metabolite abundance associated with aromatic amino acid pathways (e.g., tyrosine and tryptophan), vitamin B6 metabolism (a key cofactor for aromatic amino acid decarboxylases), and their downstream products, biogenic and trace amines, suggests a coordinated metabolic shift that may contribute to and/or amplify the FT pathophysiology.

## 1 Introduction

Fescue toxicosis (FT) is a multisystem disorder associated with ingestion of tall fescue infected with the endophytic fungus *Epichloë coenophiala* (1). This plant-fungal symbiosis enhances, through the production of secondary metabolites, grass's resistance to biotic and abiotic stressors (2). Although many of the compounds produced by the fungus play a role in plant survival and fitness, ergot alkaloids (EAs) produced by *E. coenophiala* compromise animal health, inducing fescue toxicosis (FT) (3), one of the most devastating toxicities observed in grazing livestock (4).

Ergovaline (EV) is the most abundant ergopeptine alkaloid in toxic (E+) tall fescue (5). However, the plant also contains ergoline alkaloids, such as lysergic acid (LA) and its derivatives (6). While less potent than EV, these compounds are bioactive (7), suggesting they may contribute to FT pathogenesis (8). Supporting this idea, studies have shown that animals excrete higher levels of LA despite consuming more EV (9, 10), indicating that EV is metabolized into LA or other compounds that may contribute to toxicity. This metabolic transformation is mediated, at least in part, by ruminal microorganisms (11), which are also responsible for breaking down plant material and releasing volatile fatty acids (VFAs), the main energy source for both maintenance and production (12). Thus, determining which EAs are most abundant and consistently present across multiple biological matrices will shed additional light on their metabolism under grazing conditions. Moreover, examining microbial end-products, VFAs, might reveal whether exposure to EAs, through selective microbial degradation or by EAs directly affecting rumen physiology, causes shifts in ruminal fermentation patterns.

Ergot alkaloids contribute to the complex FT pathophysiology by mimicking the action of the natural biogenic amines, serotonin, dopamine, epinephrine, and norepinephrine. Structural similarity with these molecules allows EAs to interact with their receptors in different tissues, disrupting many physiological systems (13). While the vascular system is the most studied EA target, the widespread distribution of these amines receptors suggests that EAs affect many other functions, reflecting the complex nature of this mycotoxicosis and the challenge in studying and understanding FT pathophysiology.

To better characterize the complex nature of FT, comprehensive approaches are needed. Untargeted metabolomics offers a powerful tool for exploring global metabolic alterations in response to toxic insults. By profiling a wide range of metabolites without prior bias, this technique can reveal previously unrecognized pathways and mechanisms disrupted by EAs exposure. Previous fescue grazing studies using this approach have identified alterations in lipid metabolism and aromatic amino acids (e.g., tyrosine and tryptophan) in plasma, urine and rumen fluid (14–16). These metabolic disruptions were observed in steers under continuous grazing conditions, in pastures with low EAs concentrations (16) and in pastures exceeding 1,000 ppb of EAs (14, 15). However, the effects of EAs on the metabolome of animals exposed rotationally to toxic and non-toxic fescue pastures remain unknown. This highlights the need to evaluate how this experimental rotational strategy between toxic and non-toxic pastures may affect metabolome dynamics and key signs of the disease, as well as its impact on the metabolomic profiles of other, yet underexplored, biological matrices such as saliva.

Employing high-resolution metabolomics coupled with targeted VFA analysis and integrating data across multiple biological matrices, this fall rotational grazing study aimed to: (1) characterize the metabolomic impact of EA exposure and identify potential biomarkers of exposure/effect, (2) assess metabolic recovery following removal from toxic pastures, (3) explore the dynamic of the most prevalent EAs across different biological matrices, and (4) quantify and characterize the VFA dynamics in the rumen and feces of EA-exposed steers. By integrating information across different biological matrices, this study provides a deeper and more comprehensive understanding of the metabolic effects caused by EA exposure in beef cattle rotationally grazing toxic fescue.

## 2 Materials and methods

### 2.1 Animals, treatments, and experimental design

The study was conducted in the fall of 2023 (October 18th–November 15th), on pastures located at the University of Georgia's J. Phil Campbell Natural Resources Conservation Center (Watkinsville, GA). Post-weaning Angus steers (*n* = 18; BW = 200.3 ± 4.1 kg) were blocked by weight and randomly assigned to 1.2 ha of tall fescue pastures containing a new novel endophyte (NE; Jesup MaxQ strain AR542; Max-Q, *n* = 6; 204.6 ± 7.2 kg), a toxic endophyte (E+; Jesup with wild-type endophyte, *n* = 6; 198.7 ± 8.1 kg) and endophyte-free fescue (E-; *n* = 6; 197.5 ± 7 kg). The steers were kept on these pastures for 14 days and then switched as follows: steers initially on E+ were randomly divided and reassigned to non-toxic pastures (E–, *n* = 3; NE, *n* = 3). In turn, steers initially on non-toxic pastures (E–, *n* = 3; NE, *n* = 3) were switched to E+, while the remaining animals (E–, *n* = 3; NE, *n* = 3) remained on their original non-toxic pastures. Animals remained on their newly assigned pastures for an additional 14 days. Urine, saliva, plasma, rumen fluid, and feces were collected before (pre), 2, 7, 14, 16, 21 and 28 days after pasture allocation. Fescue plants were collected on the first and the last day of the trial for endophyte and alkaloid analyses. The working area was located adjacent to the pastures where the animals were grazing. At approximately 7:15 a.m., all animals were gathered together, and samples were collected between 8:00 a.m. and 12:00 p.m. Upon completion of sampling, all animals returned together to their assigned pastures. Throughout the procedure, care was taken to ensure both operator safety and animal welfare.

### 2.2 Sample collection and processing

Blood samples were taken from the jugular vein and placed in K3 EDTA vacutainer tubes (Becton Dickenson and CO, Franklin Lakes, NJ) on ice until plasma was harvested. Fecal samples were collected by hand using fresh gloves for each collection, placed in 50 ml conical centrifuge tubes (VWR International Ltd., USA) and stored on ice. Samples of ruminal contents were collected using an oro-ruminal probe, which was washed between animals as in (17). To eliminate contamination with saliva, the first collection from each animal was discarded, and the second one was retained. Approximately 50 ml of ruminal contents were filtered using 4 layers of autoclaved cheesecloth, to separate liquid and solids fractions. A portion of the rumen fluid was immediately placed in sterile cryovial tubes and kept on dry ice. Saliva was collected using disposable swabs (Munkcare^®^) placed in the left cheek, then dragged along the sublingual area and through the oral vestibule. The collected saliva was transferred to 50-ml conical tubes (VWR) and stored on ice. Voided urine samples were collected in sterile cups, transferred to 15-ml conical centrifuge tubes (Fisher Scientific, Waltham, MA), then placed on ice. Upon arrival at the laboratory, urine and saliva samples were centrifuged (300 x *g* for 10 min at 4 °C) and aliquoted. Plasma was collected after centrifuging the blood (3,500 x *g* for 15 min at 4 °C). All the samples were stored at −80 °C until further analysis. Tiller samples were collected in Ziploc bags, protected from direct sunlight, and stored on ice. Upon arrival at the laboratory, a portion of the samples were immediately processed for endophyte detection, and the remaining material was stored at −80 °C until analysis of total ergot alkaloids.

### 2.3 Endophyte detection and total plant EAs analysis

Sampling was performed by selecting a tiller from 100 locations within the pastures, cutting the tiller at the soil surface, and transporting the samples to the laboratory. Endophyte presence was analyzed from a 3 mm cross-section of the stem base of each tiller using a commercial immunoblot test kit (Agrinostics Ltd., Co., Watkinsville, GA, USA, Cat. # ENDO797-3). In addition, total plant EAs content was determined by a second tiller cross-section using a commercial ELISA test kit (Agrinostics Ltd., Cat. # ENDO899-96p), with a limit of detection (LOD) of 1 ppb, as in (14).

### 2.4 Untargeted metabolomics sample processing

Urine, rumen fluid, plasma, and saliva samples for metabolomics were processed as described in (14). Briefly, 50 μl of sample was combined with 100 μl acetonitrile and 2.5 μl of an isotopically-labeled internal standard mixture containing [trimethyl-^13^C_3_] caffeine, [^15^N,^13^C_5_]-L-methionine, [^15^N]-L-tyrosine, [^13^C_5_]-L-glutamic acid, [^13^C_6_]-D-glucose, [3,3-^13^C_2_]-cystine, [^13^C_18_]-linoleic acid, [3',4',5'-^13^C_3_]-nicotine, [^13^C_6_]-L-histidine, and [2,3,4-^13^C_3_]-cortisol. Samples were kept on ice for 30 min before centrifugation at 20,800 x *g* for 10 min. Subsequently, 100 μl of supernatant was collected for analysis using a High-Field Orbitrap Mass Spectrometer (Thermo Fisher, Bremen, Germany) with specific instrument settings described in (18). Both reverse phase (C18) chromatography with negative electrospray ionization (ESI) and hydrophilic liquid interaction chromatography (HILIC) with positive ESI were employed, with each sample run in triplicate for both methods. Liquid chromatography-mass spectrometry conditions and methodology used were as described previously (18). Data processing involved various steps such as peak detection, noise filtering, *m/z* and retention time alignment, feature quantification, and quality filtering, executed using apLCMS v6.1.3 (19) followed by xMSanalyzer v2.0.7 (20). Data were extracted as *m/z* features, defined by *m/z*, retention time, and integrated ion intensities. Technical replicates were median summarized for subsequent bioinformatics analysis.

### 2.5 Urine, plasma, saliva and rumen fluid metabolite annotation

Metabolomics annotations presented herein were generated using either the Human Metabolome Database (HMDB); Bovine Metabolome Database (BMDB), Livestock Metabolome Database (LMDB), or the Toxic Exposome Database (T3DB). Significantly different *m/z* features were annotated against databases with a Δ 5-ppm tolerance. When the same metabolic feature was detected with different *m/z* values due to multiple co-eluting additional adducts, the feature with the lightest adduct was selected. For the negative ionization mode (C18 column), the following adducts were considered for annotation: M-H, M-H2O-H, M+Na-2H, M+Cl, and M+FA-H. For the positive ionization mode (HILIC column), adducts including M+H, M+2H, M+H+NH4, M+ACN+2H, M+2ACN+2H, M+NH4, M+Na, M+ACN+H, M+ACN+Na, M+2ACN+H, 2M+H, 2M+Na, 2M+ACN+H, M+2Na-H, M+H-H2O, and M+H-2H2O were considered.

### 2.6 Targeted VFAs analysis

A similar protocol was used for both ruminal and fecal samples (17), except that fecal samples were solubilized in distilled water (3 ml) prior to processing. Briefly, samples were centrifuged for 10 min at 10,000 × *g* and 1 ml of the resulting supernatant was mixed with 0.2 ml of a metaphosphoric acid solution (25% w/v) and frozen overnight. Samples were then thawed and centrifuged at 10,000 x *g* for 10 min. The supernatant was mixed with ethyl acetate in a 2:1 ratio of ethyl acetate to supernatant. After vortexing, 0.5 ml of the top portion was transferred to screw-thread vials for VFA analysis in a Shimadzu GC-2010 Plus gas chromatograph (Shimadzu Corporation, Kyoto, Japan) equipped with a flame ionization detector and a capillary column (Zebron ZB-FFAP; 30 m × 0.32 mm × 0.25 μm; Phenomenex Inc., Torrance, CA, USA). Sample injection volume was 1.0 μl and helium was used as the carrier gas. The column temperature was initially set at 110 °C and gradually increased to 200 °C. Injector and detector temperatures were set to 250 °C and 350 °C, respectively.

### 2.7 Metabolomics data processing and statistical analysis

The untargeted metabolic dataset was preprocessed using MetaboAnalyst 6.0 (https://www.metaboanalyst.ca) where it successfully passed the data integrity check. To enhance the quality of the dataset, noise and non-informative variables were removed using interquartile range (IQR) filtering, and features with low repeatability were excluded based on relative standard deviation (RSD/mean). Subsequently, normalization was conducted by quantile normalization, as recommended by the program's user manual for datasets exceeding 1,000 features (21), ensuring uniformity in the total ion peak area across samples. Additionally, log_10_ transformation was applied to better fit the distribution characteristics of the data, followed by Pareto scaling. Univariate and multivariate analyses were performed using metabolic features extracted from the HILIC and C18 columns. A multivariate analysis using sparse partial least squares discriminant analysis (sPLS-DA) was conducted to reduce data dimensionality and identify the features with the strongest discriminatory power among the treatment groups, applied both across the entire dataset for each 14-day period and independently at each sampling timepoint, using treatment as the grouping factor. Each features highlighted by sPLS-DA as most discriminative between E+ and other groups were analyzed further, using a script developed in the software R (version 4.4.2; R Core Team, Vienna, Austria). Day and treatment, along with their interaction (Day × Treatment), were included as fixed effects, and both factors were treated as categorical variables with sum-to-zero contrasts. To account for repeated measurements, Animal ID was included as a random effect. Models were fitted using linear mixed-effects models, and Type III ANOVA was performed to test the main effects and their interaction. When significant effects were detected, *post hoc* pairwise comparisons were conducted using estimated marginal means with Tukey adjustment for multiple comparisons.

For both 14-day periods, metabolic pathway analysis was conducted on ruminal fluid, urine, saliva and plasma high-resolution metabolomics features using mummichog 2.0 and annotated with the *Bos taurus* KEGG, and human database (22). Features differing between groups were selected for analysis based on the criteria *P* ≤ *0.05* and false discovery rate (FDR) thresholds of ≤ 0.15. Once the disrupted pathways were identified, the mean abundance of each significant metabolic feature mapped within the affected pathway was used to assess the pathway's overall trend (upward or downward). Additionally, in rumen fluid and urine, pathway analysis was performed using only the metabolic features that exhibited the specific pattern described in Section 3.2 (i.e., an increase while on E+ pastures, and return to baseline upon removal from toxic pastures.). Due to the low number of metabolic features following this pattern in saliva and blood, this analysis was restricted to rumen fluid and urine. Moreover, in the urine, several metabolic features following an inverse pattern (i.e., a decrease while on E+ pastures and increase after returning to non-toxic pastures) were detected and used for additional pathway analysis.

VFA concentrations, and ruminal pH were analyzed using a two-way repeated Analysis of Variance (ANOVA), with animal ID, treatment and day of sampling as independent variables and individual VFA concentrations as dependent variables. Graphs were generated with GraphPad Prism 5 (La Jolla, CA).

## 3 Results

### 3.1 Plant endophyte infection, total EA

Endophyte presence averaged 70% in E+, 82% in NE, and 0% in E-. Total EA in whole-plant tissue was > 6,000 ppb in E+, and 0 ppb in NE and E-.

As a separate arm of this trial, the specific EA concentration (ergovaline and lysergic acid) was also assessed in multiple biological matrices, including rumen fluid, saliva, urine, and plasma. This approach provided the opportunity to investigate the EA dynamics across different matrices and confirmed that animals were consistently exposed to these compounds throughout the study period. Moreover, environmental conditions and physiological parameters related to thermoregulation (e.g., skin surface temperature) were continuously monitored using HOBO and iButton devices, respectively. For further details, refer to Llada et al. (23).

### 3.2 Feature collection, multivariate separation across biological matrices and putative metabolites of interest

**Figure 1:**
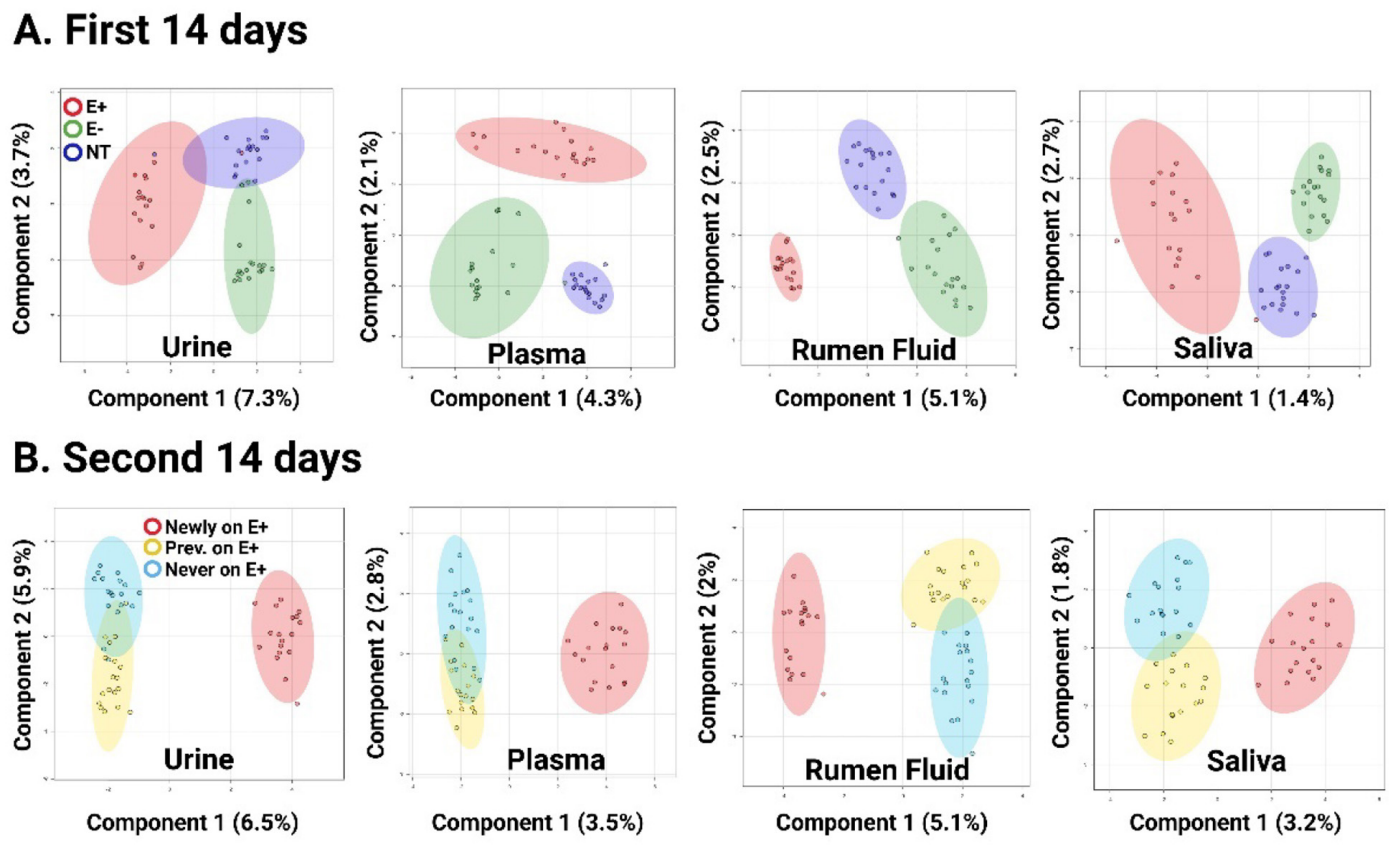
sPLS-DA (sparse partial least squares discriminant analysis) plots using the metabolic features from the urine, plasma, ruminal fluid and saliva of steers grazing toxic endophyte-infected tall fescue (E+; *n* = 6), non-toxic novel endophyte-infected tall fescue (NE; *n* = 6), or endophyte-free tall fescue pastures (E-; *n* = 6) during the first 14 days **(A)**, and **(B)** after switching pasture treatments. Each point within the sphere represents an individual sample (one animal at a single timepoint; First 14 days: days 2, 7, and 14; Second 14 days: days 16, 21, and 28).

**Figure 2:**
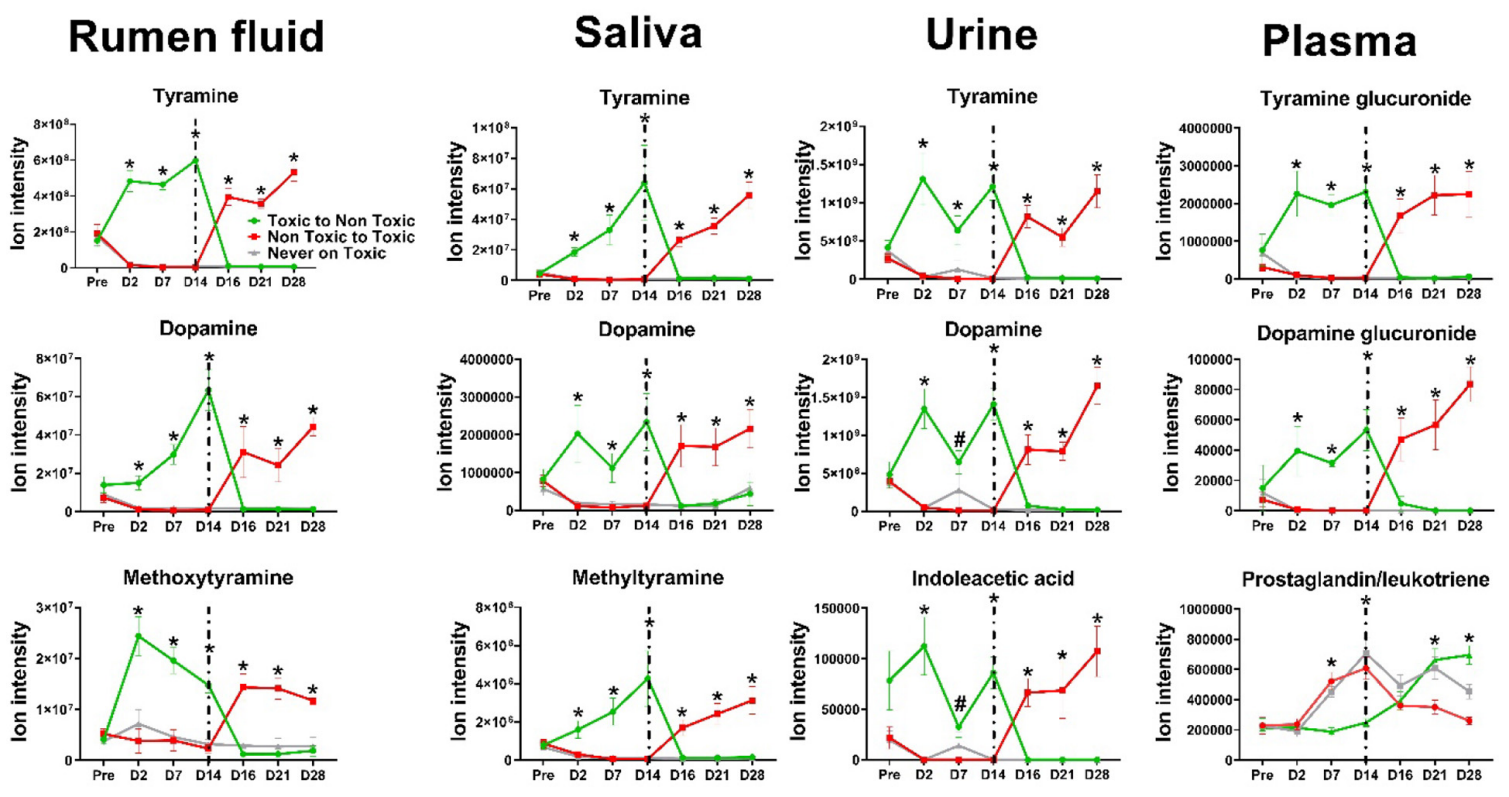
Dynamics of selected metabolic features (listed in Table 1) across different biological matrices in Angus steers during the first 14 days of grazing toxic endophyte-infected tall fescue (E+; *n* = 6), non-toxic endophyte-infected tall fescue (NE; *n* = 6), or endophyte-free tall fescue pastures (E-; *n* = 6), and after switching pasture treatments. (#) indicates a significant difference (*P* ≤ *0.05*) between the E+ group and one of the other groups, while (*) indicates a significant difference between E+ and the rest (*P* ≤ *0.05*). Data are presented as mean ± SEM; *n* = 6 animals per group. Dashed line marks the treatment switch.

After HRM processing, 16,377 unique features (i.e., metabolites with unique *m/z* and retention times) were identified in rumen fluid, 12,849 in urine, 16,022 in saliva, and 14,030 in plasma using the HILIC column. With the C-18 column, 15,477 features were found in rumen fluid, 8,842 in urine, 13,332 in saliva, and 12,332 in plasma. Using these features, a multivariate sPLS-DA analysis was applied to the entire dataset for each matrix (Figure 1A) and independently at each sampling timepoint (Supplementary Figure 1) shows day 14 post-pasture placement in both halves of the study; remaining time points are not shown, revealing clear group separation in both chromatographic columns, although it was more pronounced in the HILIC column. During the first 14 days, this separation became evident 2 days after the steers started grazing on E+ pasture and persisted until day 14 (Supplementary Figure 1), when they were switched to non-toxic pasture. During the second 14-day period, animals newly exposed to E+ showed a clear separation starting 2 days post-E+ pasture placements and continuing until the end of the study (Supplementary Figure 1). In contrast, animals previously exposed to E+ showed a similar overall metabolomics profile to those that had never grazed toxic pastures (Figure 1B). sPLS-DA analysis identified 93, 88, 66, and 60 discriminative metabolomic features in rumen fluid, urine, saliva, and plasma, respectively, distinguishing animals grazing on E+ from those in the other groups. Among the metabolic features, 59 in rumen fluid, 47 in urine, 15 in saliva, and 16 in plasma exhibited significant effects of treatment and/or day × treatment (Table 1; Figure 2), following a specific pattern. This pattern was characterized by: (A) prior to pasture placement, no differences (*P* > *0.5*) were observed in ion intensity across groups, (B) levels began to increase and reached statistical significance (*P* ≤ *0.001*) on days 2, 7, and 14 in E+ steers, (C) after switching from toxic to non-toxic pastures, levels decreased within 2 days, aligning with those observed in the negative control group, and (D) during the second 14-day period, steers newly exposed to E+ showed a similar rapid increase in the feature, which remained elevated (*P* ≤ *0.001*) until the end of the study. A Venn diagram was used to identify which of these metabolic features that followed the specific pattern were shared across biological matrices. The overlapping features that could be annotated are listed in Table 1, and selected examples are shown in Figure 2. The remaining overlapping features without annotation, along with metabolic features that followed the same pattern but did not overlap across matrices, are presented in Supplementary Tables 1, 2. Similarly, in rumen, plasma, and saliva, although fewer in number, several metabolic features following the inverse pattern (i.e., a decrease while on E+ pastures and an increase after switching to non-toxic pastures) were identified. These features, whether annotated or not, can be found in Table 1, Figure 2, and Supplementary Table 1.

**Table 1:** Metabolic features that (a) increase while on E+ pastures and return to control levels after removal, and (b) overlap across different biological matrices.

Annotation	Plasma		Rumen Fluid		Saliva		Urine		Database	Adduct
	*mz*	*Rt*	*mz*	*rt*	*mz*	*rt*	*mz*	*rt*		
Hexacosanoic acid	155.1223	36	155.1216	47	155.1217	46	155.1216	41	BMDB0002356	[M+3Na]^+^
Propionylpyrrole	124.0757	38	124.0757	36	124.0757	33	124.0757	36	HMDB32492	[M+H]^+^
Pipecolic acid	112.0764	57	112.0758	39	112.0758	37	112.0758	66	LMDB00031	[M+H–H_2_O]^+^
Tyramine glucuronide	331.1509	70	–	–	–	-	331.15	108	BMDB0010328	[M+NH_4_]^+^
L-Canaline	332.1533	85	–	–	–	-	332.1534	103	BMDB0012251	[M+ACN+Na]^+^
Dopamine glucuronide	347.1453	73	–	–	–	–	347.145	56	BMDB0010329	[M+NH_4_]^+^
Prostaglandin/Leukotriene (^*^)	333.207	78	–	–	–	–	333.207	80	BMDB0004238/ BMDB0005073	[M+H]^+^
Linolenoylglycerol	397.2594	130	–	–	–	–	397.2594	127	BMDB0011570	[M+FA–H]^−^
3-Hydroxyphenyl-valeric acid	193.0868	21	193.087	22	–	–	–	–	HMDB41666	[M–H]^−^
1H-Indole-3-carboxaldehyde	289.0981	131	289.0969	134	–	–	–	–	LMDB00661	[2M+H]^+^
N-Methyltyramine	–	–	169.1335	39	169.1335	32	169.1335	32	BMDB0003633	[M+NH_4_]^+^
Tyramine	–	–	155.1178	43	155.1179	43	155.1178	39	BMDB0004989	[M+NH_4_]^+^
Hydroxyhexanoycarnitine	–	–	138.5946	280	138.5946	273	138.5946	255	HMDB13131	[M+2H]^2^^+^
Dopamine	–	–	171.1126	130	171.1127	119	171.1127	98	BMDB0000073	[M+NH_4_]^+^
Salsolinol	–	–	197.1284	42	197.1285	40	197.1284	39	BMDB0096156	[M+NH_4_]^+^
Cyclo (leucylprolyl)	–	–	211.144	35	211.1441	30	211.1441	32	HMDB34276	[M+H]^+^
Acetyldopamine	–	–	213.1233	61	213.1234	62	213.1233	59	BMDB0096146	[M+NH_4_]^+^
25-Dimethyl-1H-pyrrole	–	–	96.0809	40	–	–	96.0809	41	HMDB32973	[M+H]^+^
Hydroxyhexanoycarnitine	–	–	276.1817	278	–	–	276.1819	249	HMDB13131	[M+H]^+^
Indoleacetic acid	–	–	97.0524	47	–	–	97.0524	43	LMDB00090	[M+H+NH_4_]^+^
Acetone	–	–	141.1023	64	–	–	141.1023	55	HMDB01659	[M+2ACN+H]^+^
Propyl alcohol	–	–	143.1178	278	–	–	143.1179	253	BMDB0000820	[M+2ACN+H]^+^
Pentadecanal	–	–	155.1479	47	–	–	155.1475	42	HMDB31078	[M+2ACN+H]^+^
L-Valine	–	–	159.1127	264	–	–	159.1128	235	BMDB000033	[M+ACN+H]^+^
Methoxytyramine	–	–	185.1284	46	–	–	185.1284	42	BMDB0012162	[M+NH_4_]^+^
Epinephrine	–	–	201.1233	60	–	–	201.1233	57	BMDB0000068	[M+NH_4_]^+^
14-Bipiperidine-1-carboxylic acid	–	–	213.1597	35	–	–	213.1598	31	HMDB60336	[M+H]^+^
Valylproline	–	–	215.139	43	–	–	215.139	55	BMDB0062269	[M+H]^+^
Phenyl-Leucine	–	–	225.1597	34	–	–	225.1598	31	BMDB0063628	[M+NH_4_]^+^
(-)-Fumigaclavine B	–	–	257.1649	29	–	–	257.1649	24	HMDB30201	[M+H]^+^
Costaclavine	–	–	241.1699	29	241.17	27	–	–	T3D3696	[M+H]^+^
Hydroxyeicosatetraenoic acids (HETEs)	–	–	-	–	172.116	118	172.1158	97	BMDB0002344	[M+H+Na]^2+^

(^*^)indicates a metabolic feature that followed the inverse pattern: decreased while on E+ pastures and increased after the switch to non-toxic pastures. Database abbreviations represent the HMDB, human metabolome database; BMDB, bovine metabolome database; LMDB, livestock metabolome database; T3DB, toxin target database.

### 3.3 Pathway analysis

**Figure 3:**
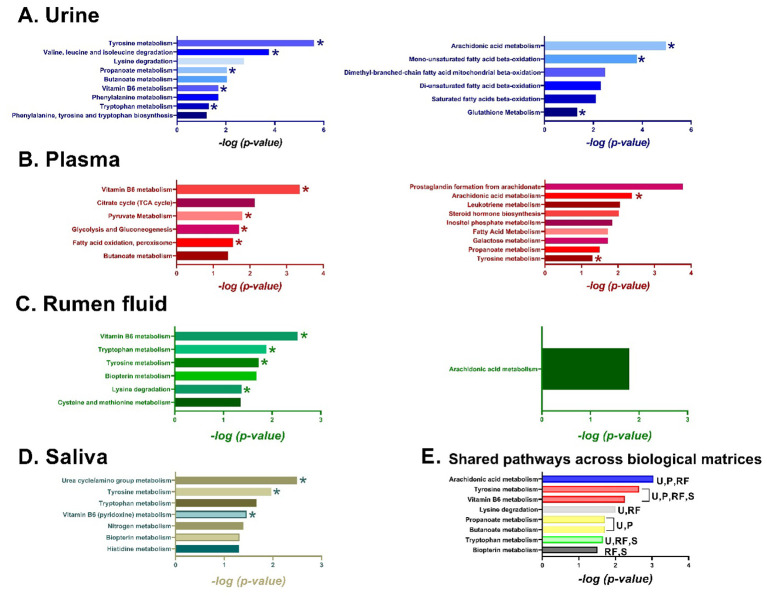
Metabolic pathways showing upward (left panel) and downward (right panel) shifts in metabolite abundance in **(A)** urine, **(B)** plasma, **(C)** rumen fluid, and **(D)** saliva of steers grazing toxic (E+) fescue. **(E)** Metabolic pathways disrupted and shared across biological matrices. An asterisk (*) indicates pathways that experienced upward or downward shifts during both the first and second 14-day periods in E+ steers; non-asterisked pathways were disrupted in only one of the two periods in E+ steers. “U”, urine; “RF”, rumen fluid; “S”, saliva; and “P”, plasma.

In urine, amino acid-related pathways, including tryptophan (P < 0.05), phenylalanine (*P* = *0.02*), and tyrosine metabolism (*P*<*0.001*), as well as lysine (*P* = *0.002*) and branched-chain amino acid degradation (*P*<*0.001*), showed an overall upward shift in metabolite abundance in E+ steers. Vitamin B6 metabolism (*P* = *0.02*) and carbohydrate-related pathways such as butanoate (*P* = *0.001*) and propanoate metabolism (*P* = *0.001*) showed the same trend. In contrast, lipid-related pathways, including fatty acid β-oxidation (*P*<*0.01*) and arachidonic acid metabolism (*P*<*0.001*), showed a downward shift. Glutathione metabolism also trended downward (*P* = *0.05*). In rumen fluid, amino acid pathways (tryptophan, *P* = *0.01*; tyrosine, *P* = *0.02*; cysteine and methionine, *P* = *0.03*; lysine degradation, *P* = *0.04*), vitamin B6 (*P* = *0.003*), and biopterin metabolism (*P* = *0.021*) showed an upward shift in metabolite abundance, while arachidonic acid metabolism followed an inverse trend (*P* = *0.01*). In saliva, amino acid-related pathways (tryptophan, *P* = *0.02*; tyrosine, *P* = *0.01*; histidine, *P* = *0.05*), nitrogen metabolism (*P* = *0.04*), the urea cycle (*P* = *0.003*), vitamin B6 (*P* = *0.03*), and biopterin metabolism (*P* = *0.05*) showed an upward shift in metabolite abundance. In plasma, carbohydrate-related pathways, including glycolysis/gluconeogenesis, butanoate metabolism, the TCA cycle, and pyruvate metabolism, showed upward shifts, as did fatty acid oxidation in peroxisomes and vitamin B6 metabolism. In contrast, lipid-related pathways, including arachidonic acid metabolism (*P* = *0.004*), fatty acid metabolism (*P* = *0.02*), steroid hormone biosynthesis (*P*<*0.01*), and leukotriene/prostaglandin metabolism (*P*<*0.001*) showed a downward shift in metabolite abundance. Additionally, inositol phosphate (*P* = *0.01*), galactose (*P* = *0.02*), and propanoate metabolism (*P* = *0.03*), also trended downward. Details about the metabolic pathways showing upward or downward shifts in metabolite abundance across the different biological matrices (A–D), as well as those shared between matrices (E), are presented in Figure 3. Among the total pathways exhibiting upward shifts in E+ steers, regardless of matrix, approximately 45% were related to amino acids, 20% to carbohydrates, 15% to vitamins, 10% to energy metabolism, 4% to lipid metabolism, and 6% to other categories. In contrast, among pathways showing downward shifts, 60% were associated with lipid metabolism, 20% with carbohydrates, 7% with amino acids, and the remaining 13% with other pathways.

In rumen fluid and urine, most of the metabolic features linked to tyrosine, tryptophan and vitamin B6 metabolism, and lysine degradation followed the same pattern: increase while on E+ and returned to control levels after switched to non-toxic pastures. In contrast, features from the arachidonic acid pathway in these two matrices and in the plasma followed the opposite pattern: decreased while on E+ and aligned with control levels after switching to non-toxic pastures.

During the second 14 days, after switching from toxic to non-toxic pasture, there were not enough significant metabolic features in any of the matrices when comparing steers previously exposed to E+ with those never exposed. This prevented pathway analysis from being performed in the steers previously exposed to toxic pasture, but it also suggests a potential rapid metabolomic recovery after removal.

Pathway analysis was also conducted using the metabolic features from rumen fluid and urine that followed the previously described pattern (increase while on toxic, decrease when switched to non-toxic). In rumen fluid, vitamin B6 metabolism was identified as the associated pathway, whereas in urine, the associated pathways included vitamin B6 metabolism, lysine degradation, and biotin metabolism. Pathway analysis using the metabolic features that followed the inverse pattern in urine (i.e., decreased while on toxic and increased after switching to non-toxic pastures) linked those features to alpha-linolenic acid metabolism and nicotinate and nicotinamide metabolism (vitamin B3), though associations were not statistically significant and showed only a trend (*P* ≥ *0.07*; data not shown).

### 3.4 EAs detected by untargeted high-resolution metabolomics

**Figure 4:**
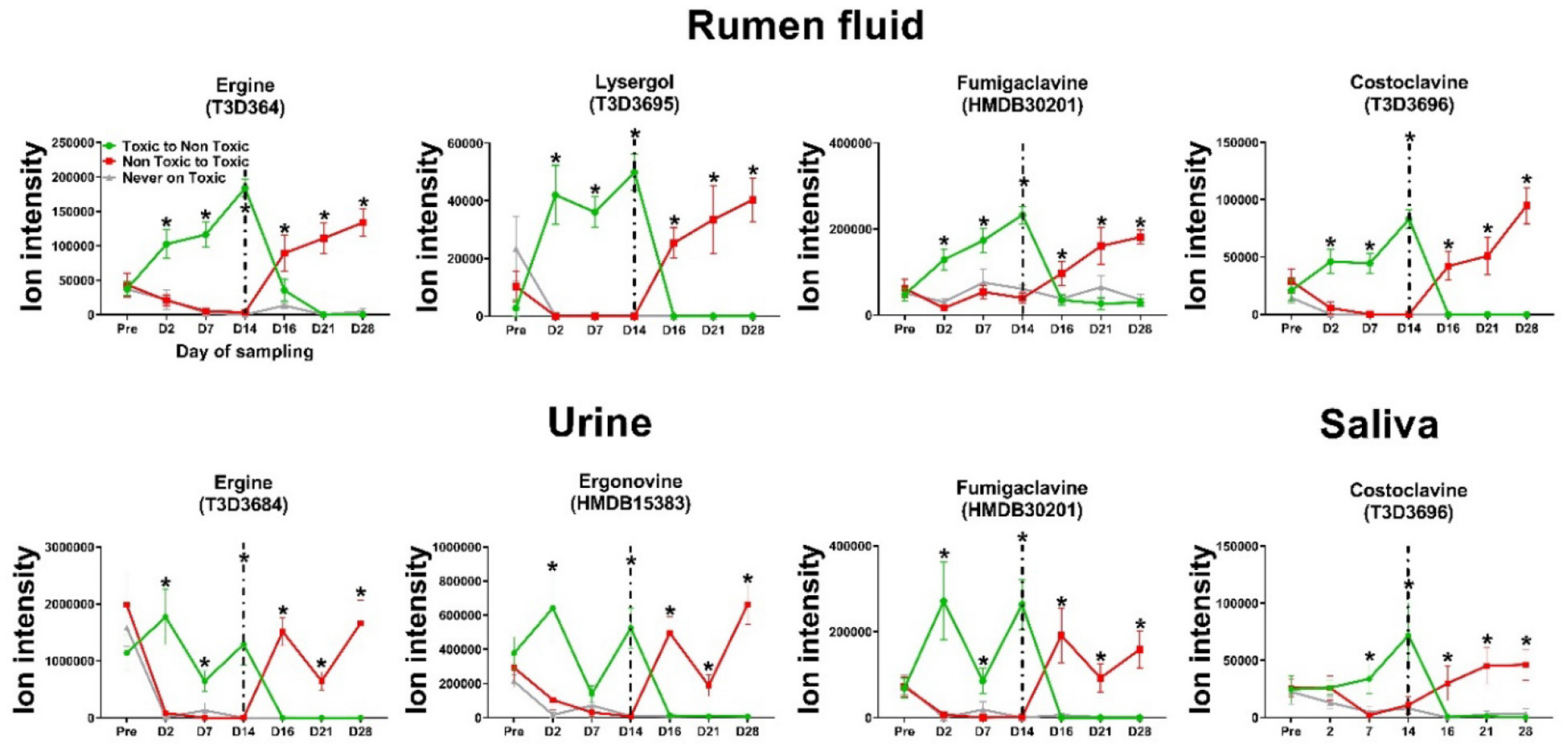
Dynamics of selected ergot alkaloids identified with untargeted metabolomics in different biological matrices of Angus steers during the first 14 days of grazing toxic endophyte-infected tall fescue (E+; *n* = 6), non-toxic endophyte-infected tall fescue (NE; *n* = 6), and endophyte-free tall fescue (E-; *n* = 6), and after switching pasture treatments. (*) indicates a significant difference between E+ and the rest (*P* ≤ *0.05*). Data are presented as mean ± SEM; *n* = 6 animals per group. Dashed line marks the treatment switch.

Multiple EAs were identified using the untargeted metabolomics approach in several biological matrices. In urine, Nicergoline (251.0793 *m/z*, 61.4 s, HMDB14837, [M+H+NH4]^+^), Ergonovine (172.1094 *m/z*, 39.2 s, HMDB15383, [M+H+NH4]^+^), Ergine (268.1769 *m/z*, 37.6 s, T3D364, [M+H]^+^), Fumigaclavine (257.1649 *m/z*, 24.4 s, HMDB30201, [M+H]^+^), and Lysergic Acid Diethylamide (362.1645 *m/z*, 63.8 s, T3D3582, [M+K]^+^) were detected. In saliva, Methylergonovine (179.1179 *m/z*, 27.6 s, HMDB14497, [M+H+NH4]^+^) and Costoclavine (241.17 *m/z*, 26.6 s, T3D3696, [M+H]^+^) were found. Rumen fluid analysis revealed Methylergonovine (179.1178 *m/z*, 36 s, HMDB14497, [M+H+NH4]^+^), Ergonovine (172.1098 *m/z*, 127.1 s, HMDB15383, [M+H+NH4]^+^), Lysergol (237.1387 *m/z*, 30.8 s, T3D3695, [M+H–H_2_O]]^+^), Ergine (268.1444 *m/z*, 31.2 s, T3D3684, [M+H]^+^), Costoclavine (241.17 *m/z*, 28.8 s, T3D3696, [M+H]^+^), Fumigaclavine (257.1649 *m/z*, 28.8 s, HMDB30201, [M+H]^+^), and Methylsergide (186.125 *m/z*, 48 s, HMDB14392, [M+H+NH4]^+^). All of them followed a similar pattern: their levels spiked within 2 days on E+ pastures, remained elevated, and returned to control levels within the same timeframe after removal from E+ pastures. No EAs were detected in plasma. Ergonovine, ergine, and fumigaclavine B were shared between rumen fluid and urine, while costoclavine and methylergonovine were shared between rumen fluid and saliva. The dynamics of selected alkaloids are illustrated in Figure 4.

### 3.5 Ruminal pH, and rumen and fecal VFAs concentrations

Ruminal pH remained unchanged throughout the study, with no significant differences observed during the first (overall mean for the three groups: 7.1; *P* > 0.8), the second 14 days (E+ steers: 7.1; other groups: 7.0; *P* > 0.5), or across sampling days (*P* > *0.2*) (data not shown).

**Figure 5:**
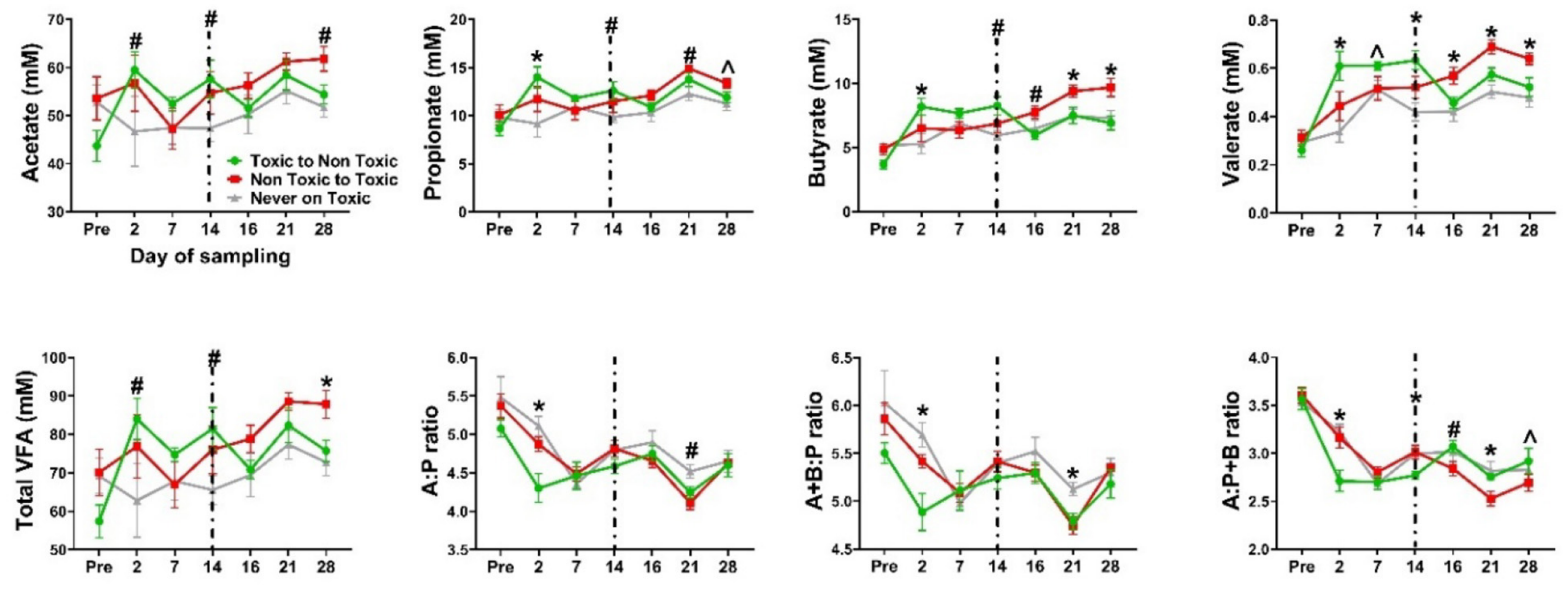
Ruminal dynamics of Volatile fatty acid (VFAs) concentration (mM) in Angus steers grazing toxic endophyte-infected tall fescue (E+; *n* = 6), non-toxic endophyte-infected tall fescue (NE; *n* = 6), or endophyte-free tall fescue (E-; *n* = 6) for 28 days with a pasture treatment switch on day 14. (#) indicates a significant difference (*P* ≤ *0.05*) between the E+ group and one of the other groups, (*) indicates a significant difference (*P* ≤ *0.05*) between the E+ group and the rest, while (Λ) indicates trends (*P*<*0.1*>*0.05*) between treatments. “A”, acetate,; “P”, propionate; “B”, butyrate. Data are presented as mean ± SEM; *n* = 6 animals per group. Dashed line marks the treatment switch.

In the rumen fluid, no differences (*P* ≥ *0.1*) were observed between groups in total or individual VFAs prior to pasture placement. During the first 14 days, the total VFAs and acetate tended (*P* ≥ *0.08*) to be higher in E+ steers, while propionate, butyrate, and valerate concentrations were significantly higher (*P*<*0.03*). Isobutyrate, isovalerate, and caproate showed no statistical differences (*P* > *0.2*) but were numerically higher in E+ steers. Within sampling day, total VFAs, acetate, propionate, butyrate, and valerate, increased after 2 days on toxic fescue and remained elevated until day 14; after switching to non-toxic pastures, levels returned to control levels, suggesting no residual effect (Figure 5). During the second 14 days, total VFAs, acetate, propionate, butyrate, valerate, and caproate concentrations were higher (*P* ≤ *0.02*) in newly exposed E+ steers than in previously exposed or never-exposed animals. Isobutyrate and isovalerate were numerically higher in newly exposed steers. Within sampling days, the VFAs that showed overall higher concentrations in steers newly exposed to toxic pastures followed the previously mentioned pattern: they increased after 2 days on E+ pastures and remained elevated until the end of the study (Figure 5).

In the fecal matter, no differences were observed in VFAs concentrations analyzed during the first (*P* ≥ *0.2*) or second 14-day study periods *(P* ≥ *0.3*) (data not shown).

## 4 Discussion

The major finding of this study is that grazing toxic fescue (E+) in the fall appears to disrupt energy metabolism in steers, shifting energy use from lipids toward amino acids and carbohydrates. This shift occurs rapidly and appears to be reversible, indicating EA-dependent metabolic disruption and recovery. Amino acids (e.g., lysine, histidine, and branched-chain amino acids) and carbohydrates pathways (e.g., gluconeogenesis, glycolysis, pyruvate metabolism, TCA cycle) showed an upward shift in metabolite abundance, whereas lipid-related pathways, including fatty acid β-oxidation, trended downward. This suggests increased reliance on protein and carbohydrate-derived substrates when lipid utilization is reduced. Upward shifts in aromatic amino acid (AAA) pathways in rumen fluid and saliva, particularly tryptophan and tyrosine, along with increased biopterin and vitamin B6 pathways activity, cofactors for biogenic amine (BAs) synthesis and bacterial AAAs decarboxylation enzymes indicate microbial contribution to the BAs and trace amines detected across matrices. Activation of the vitamin B6 pathway may also reflect responses to EA-related inflammatory or antioxidant stress. Untargeted metabolomics revealed that the primary EAs in the urine, saliva, and rumen fluid were clavine-type alkaloids and simple lysergic acid amides. This indicates that EAs undergo rumen transformation, and that metabolic and absorption processes favor simpler ergoline structures over complex ergopeptines. Although this untargeted approach is not optimized for EA detection, the results align with the targeted EA data from Llada et al. (23) and highlight the need to investigate the biological roles of simpler alkaloids in FT pathophysiology.

VFAs produced by ruminal microbes (MOs) are the primary energy source for cattle. Acetate, butyrate, and propionate are the main VFAs produced by MOs, with propionate the only glucogenic (12), while the other two provide energy for different tissues. In this study, all three VFAs and total VFA concentrations were consistently elevated in the rumen of E+ steers but returned to baseline after removal from toxic pastures, indicating that, regardless of the cause, the effect is not persistent. The higher rumen concentration observed here suggests either increased VFAs production or reduced VFAs absorption. Given that animal grazing E+ have reduced feed intakes (24), reduction in absorption seems more likely. Impaired absorption may result from decreased ruminal motility (25) or the vasoconstrictive effects of EAs on ruminal blood vessels (26), both of which would limit VFAs transport across the rumen wall. Regardless of the exact mechanism, lower gluconeogenic substrates and less energy fuel for tissues would be absorbed, raising a question: How do steers meet their energy demands under these conditions? One explanation posited based on the current data is that they may shift toward greater dependence on lipids, and/or amino acids.

Lipids are the most energy-dense nutrient for mammals, stored in adipose tissue, and mobilized during energy deficits (27). Once inside the cell, fatty acids are transported into the mitochondria via the carnitine shuttle, and oxidized through β-oxidation, the most efficient ATP-producing pathway (28, 29). In this study, lipid metabolism, including β-oxidation, shifted downward in E+ steers, accompanied by higher urinary carnitine metabolites (e.g., hydroxyhexanoylcarnitine; Table 1), indicating likely impairment of mitochondrial fatty acid oxidation. Consistent with this, previous research reported downregulation of mitochondrial function and ATP-related genes in E+ rats (30), and severe mitochondrial damage in cardiomyocytes of lambs fed E+ seed (31). Mitochondrial dysfunction increases reactive oxygen species, promoting oxidative damage. The oxidative stress-related metabolites detected in the urine of E+ steers (e.g., dityrosine, ethenodeoxyadenosine, glutarylcarnitine; Supplementary Table 1) support this and suggest that mitochondrial impairment may contribute to the energy imbalance observed here. Additionally, the upward shift in the peroxisomal β-oxidation pathway in the plasma of the E+ group may represent a compensatory response to mitochondrial dysfunction. Although mitochondria are the primary site for fat oxidation and energy production, peroxisomes act as an auxiliary system under mitochondrial impairment, oxidative stress, or lipid overload (29).

Alternatively, reduced β-oxidation may result not only from mitochondrial dysfunction but also from limited lipid mobilization, restricting substrate availability. Some EA accumulation has been reported in adipose tissue (32), where they may disrupt lipid metabolism. Previous studies found reduced blood triglycerides and cholesterol in animals exposed to EAs (33, 34). Although these metabolites were not measured here, the steroid hormone biosynthesis pathway, dependent on cholesterol, showed a downward shift in the plasma of E+ steers. In addition, bromocriptine, a synthetic EA, has also been shown to downregulate lipid-metabolism genes in mesenteric adipose tissue, potentially impairing lipid mobilization and storage (35). Similarly, steers consuming EV (2.7 ppm) did not show elevated non-esterified fatty acids before feeding, suggesting impaired fat mobilization during periods of low nutrient intake (36). Together, these findings suggest that EAs may hinder fat utilization, limiting energy availability under reduced feed intake, a common condition in animals grazing E+ (24). When lipid-derived energy is insufficient, animals may shift toward alternative substrates, prioritizing carbohydrates and amino acids. This includes activating gluconeogenesis (glucose synthesis from non-carbohydrate precursors, such as glucogenic amino acids) and glycolysis (conversion of glucose to pyruvate for rapid ATP production), both of which showed upward shifts in E+ steers. Additionally, branched-chain amino acid (BCAA: leucine, isoleucine, valine) pathways showed the same trend, and elevated urinary products of BCAA catabolism (e.g., 2-methyl-3-hydroxybutyric acid, L-leucine) were detected, suggesting that BCAAs may support glucose production. In alignment, EV-exposed rats showed upregulation of genes related to gluconeogenesis and protein catabolism (30). Further evidence of protein catabolism and/or amino acid utilization for energy production is supported by the consistent upward shift in vitamin B6 metabolism across matrices, including plasma. Vitamin B6 is a key cofactor for transamination and deamination reactions in amino acid breakdown (37); its enhanced activity likely reflects increased demand for B6-dependent enzymes during amino acid turnover. Reduction in IGF-1 (38, 39), and GASP-1 (40, 41), both important for muscle growth, have been reported in animals grazing on E+, further supporting a shift toward catabolism or dietary amino acids utilization for energy production rather than muscle accretion. The upward shift of the lysine degradation pathway in urine, along with elevated levels of pipecolic acid across matrices (Table 1) align with increased amino acid use as an energy source. As a key intermediate in lysine catabolism, pipecolic acid reflects increased lysine breakdown, a process primarily aimed at generating acetyl-CoA to fuel the TCA cycle (42). Finally, the upward shift of the Krebs cycle in the plasma of E+ steers reinforce a shift in energy use from lipids to carbohydrates and amino acids. The Krebs cycle uses Acetyl-CoA as fuel, derived from β-oxidation of fatty acids, glycolysis/pyruvate from carbohydrates, or amino acid catabolism (43). Since β-oxidation exhibited a downward shift while the Krebs cycle showed an upward shift, Acetyl-CoA is likely being sourced primarily from glucose and/or amino acids rather than fatty acids.

Metabolic pathways related to aromatic amino acid (AAAs) metabolism, including tryptophan and tyrosine, exhibited an upward shift in the rumen, saliva, and urine of E+ steers. Additionally, pathways involved in the biosynthesis of phenylalanine, tryptophan, and tyrosine followed a similar pattern in urine, indicating enhanced breakdown of these amino acids and accumulation of excreted intermediates. Previous fescue grazing studies reported alterations in these pathways (14, 16), though the mechanisms remain unclear. The tryptophan pathway contributes to serotonin synthesis, and EAs have been shown to decrease circulating serotonin (44), as well as related metabolites such as melatonin (45) in cattle. Studies in conventional mice showed higher plasma serotonin and lower serum tryptophan compared to germ-free mice (46, 47), suggesting that gut microbes consume tryptophan and convert it locally to serotonin, a mechanism potentially relevant to the rumen environment as well. Here, multiple tryptophan-derived metabolites increased in the rumen fluid of E+ steers (Supplementary Table 1), including compounds involved in serotonin biosynthesis (e.g., 5-hydroxy-L-tryptophan and serotonin), as well as others such as tryptophol, chlorotryptophan, and caffeoyltryptophan. Tryptophan-producing bacteria (e.g., *Clostridium spp*) were enriched following ergovaline (EV) administration, suggesting a role in EAs degradation (11). These microbes may consume tryptophan and its derivatives, explaining the upward trend of this pathway in the rumen and in saliva. Additionally, members of this bacteria group like *Ruminococcus gnavus* and *Clostridium sporogenes*, both found in the rumen (48, 49), possess aromatic amino acid decarboxylase enzymes (AADC), that convert tryptophan into tryptamine (50). In the gut, tryptamine stimulates enterochromaffin cells to release serotonin; however, these cells are absent in the rumen. The detection of serotonin in rumen fluid (Supplementary Table 1) therefore raises the possibility that rumen microbes may synthesize serotonin directly, independent of enterochromaffin cell signaling, though this remains to be experimentally confirmed. Parallel to these findings, metabolic features from the biopterin pathway, which produces tetrahydrobiopterin (BH_4_), a cofactor for BAs synthesis (51), showed an upward shift in the rumen and saliva of E+ steers. Though less studied in prokaryotes, this pathway exists in some microbes (52) and may aid AAAs conversion to BAs in the rumen, warranting further research. Further evidence supporting microbial involvement in tryptophan transformation includes increased indole-related metabolites, such as indole-3-carboxaldehyde in plasma and indoleacetic acid in urine of E+ steers, both microbial products of tryptophan degradation (49), which is consistent with previous reports (14). In summary, these findings suggest that grazing E+ fescue may enrich tryptophan-utilizing bacteria that consume tryptophan and promote the local synthesis of BAs, like serotonin, and other tryptophan-derived metabolites.

**Figure 6:**
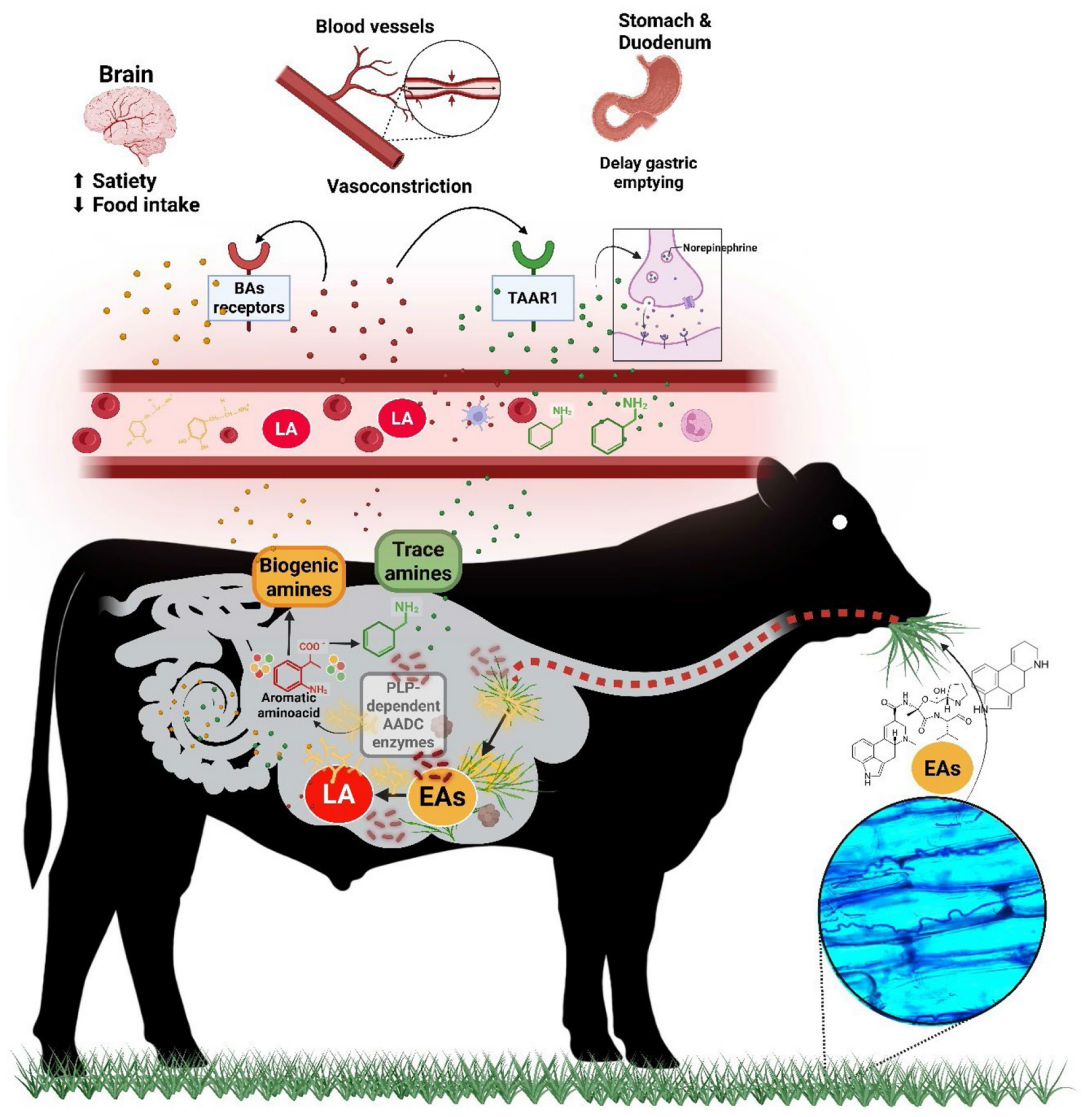
Proposed mechanism linking grazing E+ fescue to microbial-derived metabolites with potential biological effects. Once animals consume plants containing ergot alkaloids (EAs), these compounds are released and transformed by rumen microorganisms into simpler ergoline alkaloids. These same microbes, by expressing PLP-dependent AADC enzymes, can convert aromatic amino acids into bioactive compounds, such as trace amines (TAs), and biogenic amines (BAs). The simple ergoline alkaloids, together with the TAs, can be absorbed into the bloodstream. Lysergic acid (LA) acts on biogenic amine receptors and trace amine-associated receptors (TAAR1), while TAs act on TAAR1, or modulate the release and reuptake monoamines, e.g., of norepinephrine, inducing biological changes. The BAs may act locally in the gut, modulating gastrointestinal function, and a portion can be absorbed into the circulation to interact with BAs receptors systemically. PLP refers to pyridoxal phosphate (vitamin B6), and AADC stands for aromatic L-amino acid decarboxylase enzymes. Red dots represent simple ergoline alkaloids (e.g., lysergic acid), green dots represent TAs, and yellow dots represent BAs. Created with BioRender.

The tyrosine pathway exhibited an upward shift in all matrices except plasma, where it trended downward. Metabolites from this pathway, such as dopamine, trace amines (TAs) and their derivatives, were elevated in saliva, rumen, and urine of E+ steers, while their conjugated forms (e.g., dopamine glucuronide, tyramine glucuronide) increased in plasma. This raises the question: if the pathway showed a downward shift in plasma, where were these metabolites coming from? One possibility is peripheral production, such as the gastrointestinal tract. As mentioned, microbes expressing AADC enzymes can act on multiple AAAs substrates, for example, converting L-tyrosine into tyramine and L-DOPA into dopamine (53). Several *Firmicutes* genera, including *Blautia, Clostridium, Enterococcus*, and *Ruminococcus*, can produce aromatic amines through AADC activity (54). AADC enzymes are pyridoxal phosphate (PLS; Vitamin B6)-dependent (55). In this study, vitamin B6 pathway exhibited an upward shift across all matrices, possibly increasing cofactor availability and enhancing microbial decarboxylation. Additionally, some microbes, such as *Clostridium spp*, possess β-glucuronidase enzymes (56), which may explain the presence of glucuronidated metabolites in plasma. Previous fescue grazing studies identified several of these microbial taxa (57, 58), reporting shifts within the phylum *Firmicutes* in the feces of E+ steers, particularly in genera such as *Clostridium* and *Ruminococcus*. By integrating metabolomic and microbiome data from steers exposed to E+ fescue, Mote et al. (15) showed that key metabolites distinguishing E+ from control animals, particularly pyridoxal, a central component of vitamin B6 metabolism, were strongly correlated (*r* > 0.7) with bacterial OTUs across rumen fluid, rumen solids, and feces, including *Prevotella ruminicola, Clostridium*, and *Ruminococcus*. In addition, microbes capable of degrading EAs (11) also express AADC enzymes. Integrating these results with the previous paragraph provides a basis for hypothesizing that EA ingestion may enrich or stimulate the activity of microbes expressing PLS-AADC enzymes, enhancing the local production of BAs and related compounds such as TAs, which could contribute to FT (Figure 6). Moreover, given their potential microbial origin, their detection in easily accessible biological matrices, and their use as biomarkers of gut dysbiosis (59), TAs should be considered as potential biomarkers of rumen dysbiosis associated with EA ingestion.

Dopamine (DA) signaling is known to mediate some EA-induced symptoms (60, 61). In fact, the administration of a DA antagonist, improved weight gain and grazing time in E+ steers (62). In contrast, TAs' role in this mycotoxicosis is underexplored. As in another fall grazing study (16), TAs levels, especially tyramine, were elevated in all analyzed matrices in E+ steers. TAs, also referred to as “false transmitters,” are structurally similar to classic vertebrate biogenic amines (63), much like EAs. They act as indirect sympathomimetics by triggering norepinephrine release and enhancing adrenergic stimulation (64) (Figure 6). TAs also bind to trace amine-associated receptors (TAARs) (Figure 6), particularly TAAR1, a G protein-coupled receptors expressed in the cardiovascular, nervous, and GI systems, as well as in endocrine-related organs such as the pancreas (65, 66). TAAR1 activation triggers the release of hormones involved in energy homeostasis and metabolism, such as insulin, GLP-1, and PYY (65). TAAR1 is also expressed in adipose tissue, and data from rats suggest an antilipolytic effect (67, 68), which may explain the downward shift in lipid pathways observed here. Interestingly, some ergopeptines and ergoline derivates, including lysergic acid, the backbone of EAs, has also been shown to activate TAAR1 (69) (Figure 6). Regardless of the mechanism, TAs have similar effects to those observed with EAs ingestion. For example, TAs induce blood vessel contractions both in peripheral (70, 71) and core (72, 73) vascular beds, including the mesenteric arteries, aorta, coronary arteries, digital arteries, forearm vessels, among others. Moreover, studies in rats have shown that TAs reduce feed intake and delay gastric emptying, among other effects (65) (Figure 6).

Arachidonic acid (AA) pathway showed a downward shift in E+ steers, consistent with previous fescue grazing trials where AA was among the most affected pathways (58), even in pastures with low EA levels (16). In this study, metabolites annotated as prostaglandin and leukotriene decrease while on E+ and increase upon removal to non-toxic pastures, reinforcing the EA impact on this pathway. Three mechanisms may explain this downward shift. First, some fungal metabolites directly inhibit phospholipase A_2_ (PLA_2_) (74), the enzyme releasing AA from membrane phospholipids. Supporting a direct EA effect, *in vitro* data showed that ergotamine and dihydroergotamine abolished α-adrenergic stimulation of prostaglandin synthesis in cultured cells, an inhibition that disappeared when exogenous AA was supplied, suggesting that EAs may block PLA_2_ activation (75). Similarly, ergotamine also reduces AA conversion into prostaglandins and thromboxane in human platelets (76) and in guinea pig ear slices (77). Second, EA-induced vasoconstriction in core and peripheral tissues (78) may limit oxygen availability, impairing the activity of enzymes that convert AA into prostaglandins (cyclooxygenase, COX) and leukotrienes (lipoxygenase, LOX), which are oxygen-dependent (79). Vitamin B6 provides a third plausible mechanism. B6 is required for: (a) converting linoleic acid into AA (80), (b) eicosanoid synthesis (81), and (c) supporting antioxidant pathways such as glutathione (82) and vitamin B3 metabolism (83). Here, plasma vitamin B6 pathway exhibited an upward shift, while AA metabolism in plasma, and linoleic acid, vitamin B3, and glutathione metabolism in urine showed a downward shift. The downward shift of these pathways in urine likely reflects high systemic demand, potentially driven by EA-induced oxidative stress (84). However, this does not fully explain the downward shift of the plasma AA pathway, which would be expected to increase if levels of its precursor, linoleic acid, were sufficient. As mentioned, a direct EA effect on PLA_2_ may limit AA release from membranes. Additionally, as observed here, increased protein catabolism or amino acids utilization for energy production, along with elevated TAs production, both B6-dependent processes, may also increase B6 demand, overwhelming its capacity to support B6-dependent pathways, such as AA. The downward shift of these pathways in urine might also suggest lower circulating metabolite levels, but, nonetheless, the high systemic B6 demand appears to be a common factor affecting pathways that rely on it. Regardless of the mechanism, disruption of B6 pathway could reduce antioxidant capacity, promote a pro-inflammatory state, and exacerbate EA-induced oxidative stress. Moreover, the consistent disruption of AA pathway across multiple studies highlights the need to clarify the underlying mechanisms, physiological consequences and opportunities for therapeutic targeting.

The metabolic perturbations induced by exposure to toxic fescue were transient, with rapid normalization once steers were removed from E+ pastures. This conclusion is supported by sPLS-DA analysis conducted during the second period across all biological matrices, which revealed that the metabolomic profiles of previously exposed steers were similar to those never exposed. The absence of persistent disruption was reinforced by the inability to perform pathway enrichment analysis in the second period, due to the insufficient number of significant metabolic features detected in any matrix when comparing the previously exposed group to the never-exposed group. Combining the EA dynamic from this untargeted metabolomics study with our targeted ergovaline and LA results (23) indicates that E+ effects on the metabolome are transitory and EA-dependent. Overall, these findings show that EA-induced metabolic shifts are largely reversible and do not elicit a residual imbalance once the toxin (s) challenge is removed, which is important management consideration for FT.

The endophytic fungus in tall fescue produces several EAs, including ergopeptines (e.g., EV), ergolines (e.g., lysergic acid (LA) and its amides), and clavines. They all share a common ergoline ring, structurally similar to biogenic amines, necessary to elicit clinical signs of FT (85). Ergopeptines represent ~50% of plant EAs, 94% of which is EV, reason why is considered the main contributor to FT (5). However, the remaining 50% also contain the ergoline ring and should not be overlooked. Here, using an untargeted approach, ergopeptines were not detected in plasma or urine, supporting reports of limited absorption (8) and extensive ruminal transformation (86). In contrast, clavine-type alkaloids and simple LA amides were the main EAs detected in saliva, rumen fluid and urine of E+ steers. Beyond the shared ergoline structure, two mechanisms may support their contribution to FT: (a) they cross the ruminal wall more readily than ergopeptines, increasing systemic exposure (8, 87), and (b) animals excrete more ergoline alkaloids than they ingest (9, 88), suggesting a microbial transformation of EV to simple alkaloids (e.g. LA), increasing the overall simple EA burden. This microbial transformation may partially compensate for the lower vascular potency of simple ergolines compared to EV (89). Ergonovine and methylergonovine, LA-derived alkaloids, were increased in the rumen fluid, saliva, and urine. Notably, an *in vitro* study using cranial branch segments of the lateral saphenous vein from fescue-naïve cattle compared the vasoconstrictive potency of various EAs and found that ergonovine induced stronger vasoconstriction than some ergopeptine alkaloids (90). Lysergic acid amide (ergine), also elevated in the rumen fluid and urine of E+ steers, induces vasoconstriction and acts as a partial agonist or antagonist at adrenergic and serotonergic receptors (7). Lastly, contractile responses have been reported for clavines alkaloids, such as agroclavine, in the dorsal pedal vein of cattle (91) and lysergol in guinea pig iliac artery (92). Although the exact mechanism remains unclear, the latter study (92) suggests an agonist effect on 5-HT_1_-like and 5-HT_2_ receptors. Overall, simple ergoline alkaloids were the predominant EAs detected, emphasizing the importance of investigating their biological relevance and contributions to FT pathogenesis.

Valeric acid concentrations were consistently elevated in the rumen fluid of E+ steers. Besides the potential reduced absorption, this increase, though still hypothetical, may reflect microbial EAs transformation. Certain EAs, particularly ergopeptines, contain amino acids such as valine, leucine, isoleucine, and proline in their tripeptide moieties (93). Amino acid-fermenting bacteria, previously linked to EV degradation (11), utilize these amino acids as substrates and produce short-chain fatty acids as end-products. Specifically, valeric acid can result from proline fermentation (94), an amino acid present in the tripeptide moieties of all ergotoxine-type alkaloids (93). Supporting this, dipeptides containing leucine and proline (Table 1) were elevated in the rumen and saliva of E+ steers, indicating increased availability of these residues. These findings suggest that elevated ruminal valeric acid might be a product of microbial EAs transformation, though further research is needed to confirm this.

A limitation of the present study is that metabolomic profiling was performed using an untargeted approach. While providing comprehensive coverage of metabolic alterations associated with grazing E+ fescue, this type of approach is exploratory in nature. This strategy requires subsequent validation through targeted analytical methods to confirm metabolite identities (e.g., trace amines and simpler ergoline alkaloids) and obtain accurate quantification of compounds of interest. Additionally, pathway enrichment analysis, though valuable for identifying affected metabolic networks, encompasses numerous metabolites and enzymatic reactions within each pathway, thereby limiting the precision with which specific biochemical steps can be identified as drivers of the observed changes. Collectively, these results should therefore be interpreted as indicative of broad metabolic perturbations occurring under E+ grazing conditions, rather than as definitive mechanistic conclusions.

## 5 Conclusion

Grazing toxic (E+) endophyte-infected tall fescue in the fall disrupts energy metabolism in steers, shifting energy use away from lipids and toward amino acids and carbohydrates. The predominant EAs detected were clavine-type alkaloids and simple lysergic acid amides, but not ergopeptines, suggesting rumen biotransformation. An upward shift in vitamin B6 and aromatic amino acid pathways, along with increased trace and biogenic amines in the rumen, indicates that EA ingestion enriches microbial populations expressing pyridoxal phosphate (vitamin B6)-dependent aromatic amino acid decarboxylase (AADC) enzymes, converting aromatic amino acids into these bioactive compounds. By mimicking the action of biogenic amines through binding to their receptors, or by potentially activating TAAR1 receptors, these simple alkaloids alone or in combination with microbial-derived metabolites may contribute to FT pathophysiology. These findings emphasize the importance of investigating both the direct effects of simple alkaloids and the contribution of microbial-derived metabolites to the development of FT. The upward shift in vitamin B6 may also reflect a response to higher systemic demand, potentially driven by protein catabolism or amino acids utilization for energy production, as well as trace amine production, which could place constraints on B6-dependent pathways such as AA, glutathione, and vitamin B3 metabolism. Finally, the EA dynamics align with the metabolome changes onset and recovery, indicating that the negative metabolic effects of grazing E+ fescue are reversible and support rotational grazing as a toxicity mitigation strategy.

## Data Availability

The original contributions presented in the study are included in the article/Supplementary material, further inquiries can be directed to the corresponding author.
