# Supplementary material for: Metabolomic shifts in beef steers rotationally grazing toxic endophyte-infected tall fescue under fall conditions
Source: Front Vet Sci. 2026 May 15;13:1785530. doi: 10.3389/fvets.2026.1785530 (PMC13219835; doi:10.3389/fvets.2026.1785530)
Supplement: Supplementary file 1 [file Table_1.docx]

**Table 1:** Metabolic features that a) increase while on E+ pastures and returned to control levels after removal.

| Urine | | | | | | |
| --- | --- | --- | --- | --- | --- | --- |
| *mz* | | **Retention time**  **(s)** | **Annotation** | **Adduct** | **Database** | **Major Pathway / Group** |
| 156.1211 | | 42 | 12-Hydroxy-12-octadecanoylcarnitine | [M + 2H + Na]³⁺ | LMDB00939 | Fatty Acid Metabolism (Carnitine) |
| 157.0971 | | 98 | 3-Hydroxypropanal | [M + 2ACN + H]⁺ | HMDB03453 | Lipid Metabolism (Aldehyde) |
| 169.1378 | | 32 | Nonacosanoic acid | [M + 3Na]³⁺ | BMDB002230 | Lipid Metabolism (Fatty Acid) |
| 170.1306 | | 32 | DiHETrE | [M + 2H]²⁺ | BMDB0002311 | Lipid Metabolism (Eicosanoid) |
| 173.1129 | | 94 | 11,14,17-Eicosatrienoic acid | [M + H + K]^2^⁺ | LMDB00705 | Lipid Metabolism (Fatty Acid) |
| 212.1475 | | 33 | DHAP(18:0e) | [M + 2H]²⁺ | HMDB11142 | Lipid Metabolism (Ether Lipid) |
| 317.1706 | | 64 | Glutarylcarnitine | [M + ACN + H]⁺ | LMDB00766 | Fatty Acid Metabolism (Carnitine) |
| 369.1062 | | 105 | DHAP(10:0) | [M + 2Na – H]⁺ | BMDB0011675 | Lipid Metabolism (Glycerolipid) |
| 369.1307 | | 19 | DHAP(10:0) | [M + FA − H]⁻ | BMDB0011675 | Lipid Metabolism (Glycerolipid) |
| 445.139 | | 93 | 5(6)-Epoxy Prostaglandin E1 | [M + 2K - H]⁺ | BMDB0012110 | Lipid Metabolism (Eicosanoid) |
| 128.0945 | | 92 | L-Leucine | [M + 3ACN + 2H]²⁺ | LMDB00215 | Amino Acid Metabolism |
| 163.0402 | | 20 | Phenylpyruvic acid | [M−H]⁻ | HMDB00205 | Amino Acid Metabolism (Phenylalanine) |
| 187.1076 | | 63 | Alanyl-Proline | [M + H]⁺ | HMDB28695 | Peptide Metabolism |
| 193.0947 | | 95 | Pipecolic acid | [M + ACN + Na]⁺ | LMBD00031 | Amino Acid Metabolism (Lysine) |
| 230.1863 | | 48 | N6,N6,N6-Trimethyl-L-lysine | [M + ACN + H]⁺ | LMDB00319 | Amino Acid Metabolism (Lysine) |
| 252.0826 | | 60 | Methionyl-Cysteine | [M + NH₄⁺ - H₂O] | BMDB0063999 | Peptide Metabolism |
| 268.1769 | | 38 | Histidylleucine | [M + NH₄⁺ - H₂O] | BMDB0063926 | Peptide Metabolism |
| 282.1926 | | 33 | L-Gizzerosine | [M + ACN + H]⁺ | HMDB39160 | Amino Acid Metabolism (Histamine) |
| 292.1306 | | 37 | Glutaminyl-Phenylalanine | [M−H]⁻ | HMDB28804 | Peptide Metabolism |
| 306.1462 | | 39 | Ala-Phe-Ala | [M−H]⁻ | BMDB0062549 | Peptide Metabolism |
| 383.123 | | 95 | Dityrosine | [M + Na]⁺ | HMDB06045 | Amino Acid Metabolism (Tyrosine) |
| 298.092 | | 33 | Ethenodeoxyadenosine | [M + Na]⁺ | BMDB0001786 | Nucleotide Metabolism |
| 118.0591 | | 18 | 2-Methyl-3-hydroxybutyric acid | [M−H]⁻ | HMDB00354 | Organic Acids Metabolism |
| 119.0503 | | 22 | 2-Hydroxy-3-methylbutyric acid | [M−2H]²⁻ | HMDB00407 | Organic Acids Metabolism |
| 158.0462 | | 18 | Succinic acid | [M−H]⁻ | HMDB0000254 | TCA Cycle |
| 154.1056 | | 94 | Retinal | [M + H + Na]²⁺ | BMDB0001358 | Vitamin A Metabolism |
| 435.1095 | | 95 | Mesohydroxy uroporphyrin III | [M + H + Na]⁺ | BMDB0003327 | Heme / Porphyrin Metabolism |
| 441.1434 | | 88 | Dihydrocortisol | [M + 2K - H]⁺ | LMDB00423 | Steroid Hormone Metabolism |
| 289.0391 | | 20 | 4-Hydroxy-5-(3-hydroxyphenyl)-valeric acid-3-O-sulphate | [M−H]⁻ | HMDB30152 | Xenobiotic Metabolism / Phenolic |
| 331.0628 | | 17 | Aflatoxin B1 | M+F | BMDB0006552 | Mycotoxin (Xenobiotic) |
| 430.1255 | | 103 | Dopaxanthin quinone | [M + ACN + H]⁺ | BMDB0012220 | Pigment / Quinone |
| 142.11 | | 230 | NA | - | - | - |
| 171.1039 | | 93 | NA | - | - | - |
| 227.0617 | | 57 | NA | - | - | - |
| 323.0491 | | 94 | NA | - | - | - |
| 328.206 | | 93 | NA | - | - | - |
| 342.2219 | | 91 | NA | - | - | - |
| 385.101 | | 56 | NA | - | - | - |
| 395.1042 | | 32 | NA | - | - | - |
| 409.1011 | | 33 | NA | - | - | - |
| 443.1431 | | 93 | NA | - | - | - |
| 495.1826 | | 56 | NA | - | - | - |
| 570.09 | | 60 | NA | - | - | - |
| Rumen fluid | | | | | | |
| 214.153 | 21 | | 3-Oxododecanoic acid | [M−H]⁻ | HMDB10727 | Lipid Metabolism (Fatty Acid) |
| 227.0754 | | 33 | Caprylic acid | [M + 2ACN + H]⁺ | LMDB00164 | Lipid Metabolism (Fatty Acid) |
| 249.1707 | | 275 | LysoPE(18:1(11Z)/0:0) | [M + H + NH₄]⁺ | BMDB0011505 | Lipid Metabolism (Phospholipid) |
| 335.0666 | | 32 | DHAP(8:0) | [M + K]⁺ | BMDB0011685 | Lipid Metabolism (Glycerolipid) |
| 353.2335 (*) | | 22 | Prostaglandin | [M - H]- | BMDB0001442 | Lipid Metabolism (Eicosanoid) |
| 365.1971 (*) | | 21 | Leukotriene | [M - H]- | HMDB06059 | Lipid Metabolism (Eicosanoid) |
| 293.1396 (*) | | 20 | Tocopheronic acid | [M−H]⁻ | HMDB30555 | Lipid Metabolism (Vitamin E metabolism) |
| 283.1342 (*) | | 22 | Estrone-2,3-quinone | [M−H]⁻ |  | Steroid metabolism (Estrogen metabolism) |
| 156.1211 | | 47 | 12-Hydroxy-12-octadecanoylcarnitine | [M + 2H + Na]³⁺ | LMDB00939 | Fatty Acid Metabolism (Carnitine) |
| 183.1128 | | 57 | L-Phenylalanine | [M + NH₄]⁺ | LMDB00069 | Amino Acid Metabolism |
| 195.0934 | | 22 | Serotonin | M+F | BMDB0000259 | Neurotransmitter Metabolism |
| 199.144 | | 38 | 3,4-Dimethoxyphenylethylamine | [M + NH₄]⁺ | BMDB0096131 | Neurotransmitter / Alkaloid |
| 239.0833 | | 22 | 5-Hydroxy-L-tryptophan | M+F | T3D4298 | Amino Acid Metabolism (Tryptophan) |
| 317.0898 | | 46 | Tyrosyl-Proline | [M + K]⁺ | BMDB0064124 | Peptide Metabolism |
| 332.1041 | | 135 | Glycogen | [2M-H]⁻ | BMDB0000757 | Carbohydrate Metabolism |
| 226.0954 | | 17 | methyltetrahydropterin | [M+FA-H]⁻ | BMDB0002249 | Cofactor Metabolism (Folate) |
| 226.1631 | | 33 | Ercalcitriol | [M+H+Na]⁺ | BMDB0006225 | Vitamin D Metabolism |
| 379.0675 | | 134 | N1-(5-Phospho-a-D-ribosyl)-56-dimethylbenzimidazole | [M + Na − 2H]⁻ | HMDB03882 | Cofactor Metabolism (Vitamin B12) |
| 295.067 | | 16 | L-2-Hydroxyglutaric acid | [2M-H]⁻ | BMD00000694 | Organic Acid Metabolism |
| 108.0809 | | 36 | Benzylamine | [M + H]⁺ | BMDB0062051 | Amine Metabolism |
| 172.1098 | | 127 | Ergonovine | [M + H + NH₄]⁺ | HMDB15383 | Alkaloid / Mycotoxin |
| 214.1631 | | 35 | 14-Bipiperidine-1-carboxylic acid | [M + H]⁺ | HMDB60336 | Xenobiotic / Alkaloid |
| 223.0761 | | 18 | Equol | [M-H2O-H] | BMDB0002209 | Phytoestrogen (Xenobiotic) |
| 237.1387 | | 31 | Lysergol | [M + H − H₂O]⁺ | T3D3695 | Alkaloid / Mycotoxin |
| 268.1445 | | 31 | Ergine | [M + H]⁺ | T3D364 | Alkaloid / Mycotoxin |
| 271.1441 | | 27 | 2-Phenylacetamide | [2M + H]⁺ | BMDB0010715 | Xenobiotic Metabolism |
| 295.0484 | | 93 | 1234-Tetrahydro-b-carboline-13-dicarboxylic acid | [M+Cl]⁻ | HMDB32102 | Alkaloid |
| 319.0708 | | 35 | Melanin | [M + H]⁺ | HMDB04068 | Pigment |
| 338.123 | | 118 | 5-Hydroxytryptophol glucuronide | [M−H]⁻ | HMDB13200 | Tryptophan Metabolism / Conjugate |
| 359.0411 | | 16 | N-(Carbomethoxyacetyl)-4-S-chlorotryptophan | [M + Na − 2H]⁻ | HMDB30398 | Tryptophan Metabolite / Xenobiotic |
| 366.1178 | | 118 | N-Caffeoyltryptophan | [M−H]⁻ | HMDB29830 | Phenolic Compound / Conjugate |
| 435.0541 | | 69 | 2-(Methylthio)ethyl glucosinolate | [M + ACN + H]⁺ | HMDB38408 | Glucosinolate (Plant Metabolite) |
| 529.2514 | | 30 | Fosfatidilinositol (PIP) |  | BMDB009994 | Lipid Metabolism (Phospholipid) |
| 125.0892 | | 274 | NA | - | - | - |
| 197.0709 | | 30 | NA | - | - | - |
| 210.0689 | | 16 | NA | - | - | - |
| 251.118 | | 32 | NA | - | - | - |
| 254.1288 | | 33 | NA | - | - | - |
| 295.0724 | | 62 | NA | - | - | - |
| 312.0837 | | 134 | NA | - | - | - |
| 319.0721 | | 82 | NA | - | - | - |
| 334.1278 | | 148 | NA | - | - | - |
| 336.0989 | | 131 | NA | - | - | - |
| 387.06 | | 86 | NA | - | - | - |
| 440.9994 | | 47 | NA | - | - | - |
| 451.1174 | | 142 | NA | - | - | - |
| 453.1352 | | 133 | NA | - | - | - |
| 469.1291 | | 120 | NA | - | - | - |
| 482.9763 | | 55 | NA | - | - | - |
| 503.0413 | | 70 | NA | - | - | - |
| 530.2379 | | 162 | NA | - | - | - |
| 546.8789 | | 59 | NA | - | - | - |
| 557.3762 | | 22 | NA | - | - | - |
| Saliva | | | | | | |
| 156.1212 | | 45 | All-trans-13,14-dihydroretinol | [M+H+Na]⁺ | BMDB0011618 | Vitamin A Metabolism |
| 212.0852 | | 30 | Ergothioneine | [M + H − H₂O]⁺ | HMDB03045 | Amino Acid Derivative / Antioxidant |
| 370.058 | | 32 | Phenylalanylglutamine | [M + 2K + H]^3^⁺ | BMDB0064021 | Peptide Metabolism |
| 367.0397 | | 20 | Argininosuccinic acid | [M+2K-H]^+^ | T3D4294 | Urea Cycle |
| 384.0383 | | 20 | NA | - | - | - |
| 243.1419 (*) | | 142 | NA | - | - | - |
| Plasma | | | | | | |
| 156.1217 | | 38 | 12-Hydroxy-12-octadecanoylcarnitine | [M + 2H + Na]³⁺ | LMDB00939 | Fatty Acid Metabolism (Carnitine) |
| 172.1088 | | 80 | 23-Dinor-TXB2 | [M + 2H]²⁺ | HMDB02904 | Lipid Metabolism (Eicosanoid) |
| 172.1161 | | 62 | 12-HETE | [M+H+Na]^2^⁺ | LMBD00716 | Lipid Metabolism (Eicosanoid) |
| 193.0953 | | 57 | D-Pipecolic acid | [M + ACN + Na]⁺ | BMDB0005960 | Amino Acid Metabolism (Lysine) |
| 158.1005 | | 70 | 7-(Methylthio)heptanenitrile | [M + H]⁺ | HMDB31890 | Sulfur Metabolism / Nitrile |
| 186.1317 | | 179 | 9-(Methylthio)nonanenitrile | [M + H]⁺ | HMDB38437 | Sulfur Metabolism / Nitrile |
| 155.1142 | | 36 | NA | - | - | - |
| 369.0552 | | 23 | NA | - | - | - |

Pathway/group assignments are based on the primary biological role of each metabolite. 'NA' indicates metabolites with no annotation; for these entries, the pathway is reported as 'Unknown'.

*m/z* values followed by an asterisk in parentheses (*) refer to those metabolic features that followed the inverse pattern: reduced while on E+ pastures and increased after the switch to non-toxic pastures.

| Table 2: Metabolic features that overlap across different biological matrices but remained unannotated | | | | | | | | |
| --- | --- | --- | --- | --- | --- | --- | --- | --- |
| Annotation | Plasma | | Rumen Fluid | | Saliva | | Urine | |
|  | *mz* | *rt* | *mz* | *rt* | *mz* | *rt* | *mz* | *rt* |
| NA | 465.1308 | 22 |  |  |  |  | 465.1308 | 23 |
| NA | 170.1368 | 32 | 170.1368 | 40 |  |  |  |  |
| NA |  |  | 155.0015 | 47 |  |  | 155.0019 | 42 |
| NA |  |  | 157.118 | 46 |  |  | 157.1181 | 42 |
| NA |  |  | 155.2341 | 48 |  |  | 155.2338 | 42 |
| NA |  |  | 169.1292 | 34 |  |  | 169.1291 | 32 |
| NA |  |  | 269.1285 | 32 |  |  | 269.1287 | 29 |
| NA |  |  | 310.0719 | 90 |  |  | 310.0723 | 81 |
| NA |  |  |  |  | 172.116 | 118 | 172.1158 | 97 |

'NA' indicates metabolites with no annotation.

| 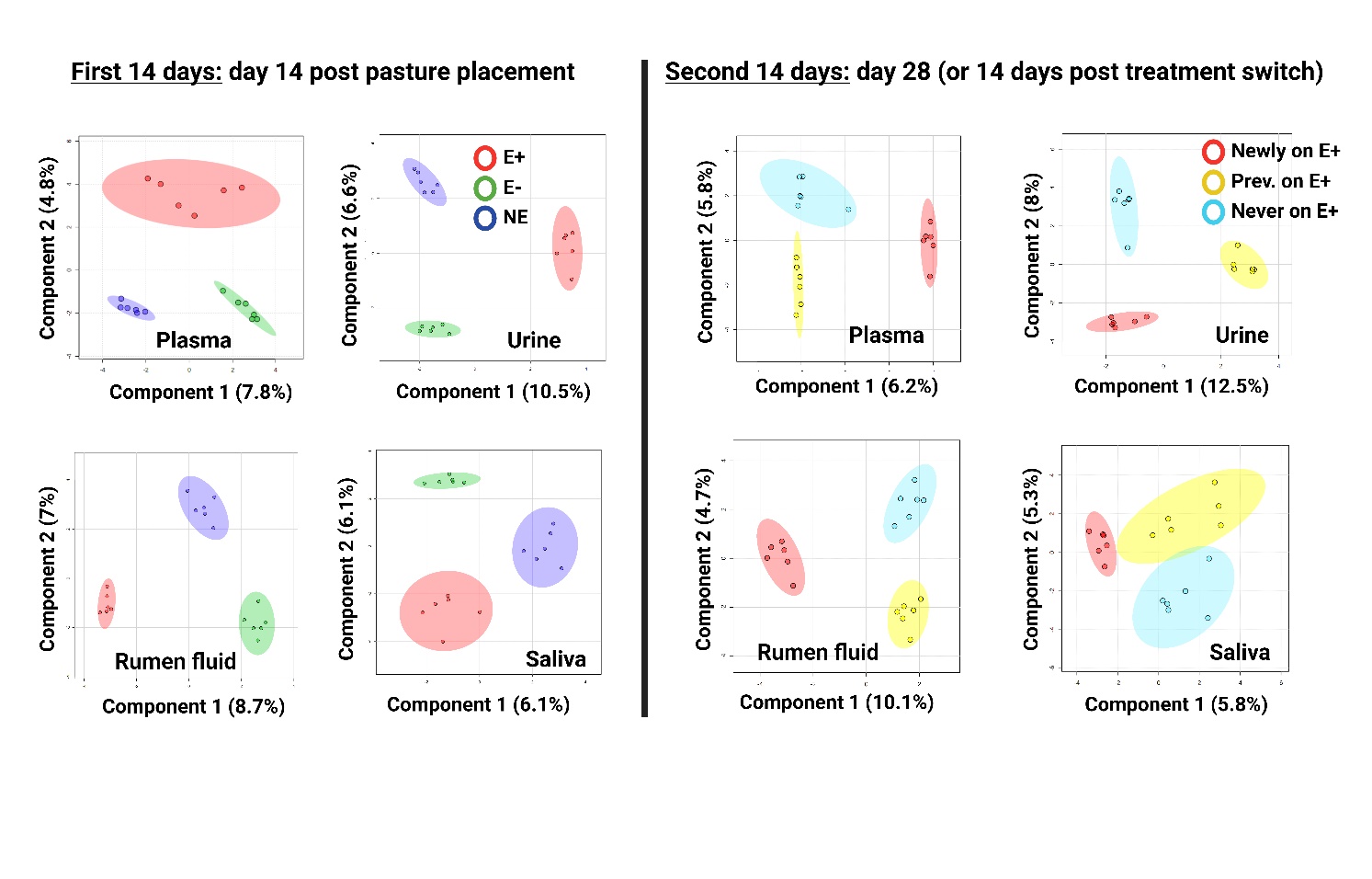 |
| --- |
| **Supplementary Figure 1:** sPLS-DA (Sparse Partial Least Squares Discriminant Analysis) plots using metabolic features from plasma, urine, ruminal fluid, and saliva of steers at 14 days post-pasture placement in the first (left panel) and second (right panel) halves of the study. |
